# Associations Among Health Insurance Type, Cardiovascular Risk Factors, and the Risk of Dementia: A Prospective Cohort Study in Korea

**DOI:** 10.3390/ijerph16142616

**Published:** 2019-07-23

**Authors:** So-Hyun Moon, Hyun-Ju Seo, Dong Young Lee, Seong Min Kim, Jeong Min Park

**Affiliations:** 1 Department of Nursing, College of Medicine, Chosun University, Gwangju 61452, Korea; 2 Department of Psychiatry, Seoul National University Hospital and Seoul Metropolitan Center for Dementia (SMCD), Seoul 03080, Korea; 3 Department of Nursing Science, Jeonbuk Science College, Jeollabuk-do 56204, Korea; 4 Department of Nursing Science, Nambu University, Gwangju 62271, Korea

**Keywords:** social class, risk factor, dementia, aged, association

## Abstract

Due to an aging population, dementia incidence has rapidly increased in South Korea, heaping psychological and economic burdens upon families and the society. This study was aimed at investigating the associations of health insurance type and cardiovascular risk factors with the risk of dementia. The study was performed using data from 15,043 participants aged 60 years and above, enrolled in the Seoul Dementia Management Project in 2008 and followed up until 2012. Factors such as demographic data, health insurance type, lifestyle factors, and cardiovascular risk factors were subjected to Cox proportional hazard regression analysis to identify their associations with dementia incidence. During the follow-up, 495 participants (3.3%) developed dementia. Medical Aid beneficiaries were associated with an increase in the risk of dementia (hazard ratio [HR] 1.77, 95% confidence interval [CI] 1.421–2.215). Upon analyzing a composite cardiovascular risk score derived from all five cardiovascular risk factors, the risk for dementia incidence in participants increased from 1.56 for the presence of three risk factors to 2.55 for that of four risk factors (HR 2.55, 95% CI 1.174–5.546), compared with those who had no risk factors. The Medical Aid beneficiaries of health insurance type and the presence of multiple cardiovascular risk factors were found to be associated with a higher risk of dementia incidence.

## 1. Introduction

The incidence of dementia in Korean people aged 65 years or older was estimated to reach 705,473 (10.0%) in 2017 and is expected to increase to approximately 2.71 million (15.0%) by 2050 [1]. Therefore, in 2017, the government of South Korea announced that a dementia policy would be implemented through the dementia national responsibility system as a national task [2]. In Korea, the total cost of managing dementia is predicted to increase from approximately 13.2 trillion won in 2015 to 106.5 trillion won in 2050, which is 3.8% of gross domestic product (GDP) [3]. Moreover, dementia will impose a considerable long-term burden on the public health care system [4]. As early detection of dementia can delay its development, and ongoing treatment can reduce socioeconomic costs, investigating dementia incidence and managing the associated risk factors are recent areas of public health focus [5].

The risk factors for dementia include genetic factors, socioeconomic factors, lifestyle factors, and vascular factors [6]. Particularly, cardiovascular risk factors are reported to play a crucial role in dementia incidence later in life [7,8]. A cohort study has reported that smoking, hypertension, hypercholesterolemia, and diabetes increase the incidence of dementia by 20–40% [9]. High cholesterol levels are reported to be strongly associated with dementia risk, probably due to the increased production of β-amyloid or the presence of apolipoprotein E type 4 allele [10]. Elevated blood pressure levels and a history of cerebrovascular disease are strongly associated with long-term dementia risks and cognitive impairment. Blood pressure-lowering interventions may reduce the risk of cognitive impairment, either by directly preventing cerebrovascular disease or by indirectly influencing the clinical expression of neurodegenerative processes [11]. According to one study, dementia risk increased from 1.27 in participants with one risk factor to 2.37 in those with all four risk factors, when compared with participants with no risk factors [9].

Previous empirical studies have suggested that health behavior differed with socioeconomic position [12]. Therefore, the relationship between socioeconomic factors and cognitive function in later life may be determined by cognitive reserve, which refers to an array of factors that provide resilience against neuropathological damage [13] and/or the differences in health behaviors [14]. Socioeconomic disadvantage was reported to be associated with a higher dementia risk, but the mechanism through which socioeconomic factors affect dementia risk is unclear. In an analysis adjusted for sociodemographic factors, an association between midlife occupational position and dementia risk has been observed, with low occupation (occupation categorized as low, intermediate, and high to represent hierarchy in the socioeconomic marker) associated with increased dementia risk (hazard ratio [HR] 2.03, 95% confidence interval [CI] 1.23–3.36) [15]. In the Framingham Heart Study investigating temporal trends in the incidence of dementia over three decades, risk reduction was observed only among participants who had at least a high school diploma (HR 0.77, 95% CI 0.67–0.88) [16]. A meta-analysis of 31 studies on incident Alzheimer’s disease yielded a pooled relative risk (RR) for low education (RR = 1.99; 95% CI 1.30–3.04) [17].

Significant differences in education level and medical insurance service type, with respect to dementia occurrence, have been observed in a study performed on elderly people with dementia, aged 60 years and above, in South Korea [18]. Age, education, exercise, and body mass index (BMI) have been identified as factors affecting dementia risk in adults aged 60 years and above [19]. These studies had a cross-sectional design, and long-term cohort follow-up studies were stated to be necessary to identify the risk factors related to incident dementia [18].

The cardiovascular risk model has been widely accepted as a primary preventive tool for cardiovascular diseases in asymptomatic adults [20,21], and it was found to be associated with dementia prevalence. This conclusion was based on data from Western populations and its appropriateness in Asian populations is limited [22,23]. Previous studies investigating the association between socioeconomic factors, income level especially, risk factors for cardiovascular disease, and dementia in the Korean population are unavailable.

Therefore, the purpose of this study was to investigate the relationship between dementia risk and demographic factors, health insurance type (reflecting economic status), lifestyle factors, and cardiovascular risk factors in a community-based general population in Korea, aged 60 years and above.

## 2. Materials and Methods

### 2.1. Participants and Data Collection

Adults aged 60 years and above, from 25 districts of Seoul Metropolitan City, screened for dementia at a public community health center and included in the Seoul Dementia Management Project (SDMP) (launched in 2007) online database, were considered for this study. Participants who were not diagnosed with dementia at baseline in the screening were invited to participate in the study [24]. Overall, 15,043 adults aged 60 years or above in January 2008 enrolled in the study voluntarily and were followed up until December 2012.

At the baseline, the dementia screening was conducted using a mini-mental status examination (MMSE-KC) [24]. Participants who obtained a mean score ≥−1.5 standard deviation (SD) from healthy elderly participants’ normative values were considered to be cognitively impaired [18]. Psychiatrists, neurologists, or community nurses trained by clinical psychologists performed neuropsychological assessments using the Korean version of the Consortium to Establish a Registry (CERAD) neuropsychological assessment battery for Alzheimer’s disease [24] or the Seoul Neuropsychological Screening Battery (SNSB) [25]. At each follow-up examination (every year or every second year after enrolment), trained psychiatrists or neurologists screened the participants who were not diagnosed with dementia at baseline for incident dementia. Additional neuropsychological testing was performed on the participants by psychiatrists or neurologists using criteria from the fourth edition of the Diagnostic and Statistical Manual of Mental Disorders [26].

We analyzed the data from the online database (DB) of Seoul Metropolitan City’s dementia management program. Each participant was asked to select “consent” in the DB, and the informed consent form was printed, signed by the participant, and retained at the local dementia support center. This study was approved by the Seoul University Medical School and the Seoul University Hospital institutional review board (No. H-1210-064-434).

### 2.2. Baseline Measurements

Age, gender, education, and cohabiting status of the participants were obtained, and BMI data were collected via self-reports or physical measurements. Health insurance type was considered as a crude socioeconomic status indicator, based on the national health insurance contribution [27]. According to the health insurance type, participants were classified into Health Insurance beneficiaries and Medical Aid beneficiaries. The Korean national health insurance (NHI) system covered the entire Korean population in July 1989 [28], and approximately 96% of South Korea’s population in 2007. The remaining 4% were covered by a separate program called Medical Aid, which is a public assistance program targeted at financially backward individuals unable to afford health care coverage [29].

Participants were questioned about their drinking habits and smoking status. Accordingly, they were classified into current drinkers and non-drinkers (those who never drank and those who had quit drinking currently). They were also classified into non-smokers, current smokers, and ex-smokers (those who had smoked in the past but had quit smoking). Participants completed self-administered questionnaires on comorbidities or risk factors related to cardiovascular diseases. The presence of known risk factors for cardiovascular disease such as hypertension, diabetes mellitus, stroke, coronary artery disease, and current smoking status, was evaluated using a composite cardiovascular disease risk score calculated by adding 1 to the score if a factor was present, and 0 if not detected. Participants with no cardiovascular risk factors were assigned a score of zero, those with one risk factor were assigned a score of one, and likewise, with five being the maximum score.

### 2.3. Data Analysis

Participants’ demographic factors, BMI, lifestyle factors, and cardiovascular risk factors were analyzed using descriptive statistics. BMI data were collected via self-reports and physical measurements. BMI was analyzed using categorical variables (reference values: <18.5 kg/m^2^ [underweight], 18.5–23 kg/m^2^ [normal], 23–25 kg/m^2^ [overweight], ≥25 kg/m^2^ [obese]) [30].

To analyze the differences in baseline characteristics between participants who developed dementia and those who did not during the follow-up period, the *χ*^2^ test was performed for categorical variables, and Student’s *t*-tests for continuous variables.

The association between the presence of cardiovascular risk factors and dementia occurrence was assessed using binary logistic regression, controlling for age. Cox proportional hazards regression analysis, adjusted for variables such as age, education status, body mass index, and smoking status, which were statistically significant in the univariate analysis, was carried out to examine the relationship between health insurance type, cardiovascular risk factors, and dementia incidence. The results of the analysis were presented as hazard ratios (HR) and 95% confidence intervals (CI). The proportional hazards assumption was justified, since the test was not significant (*χ*^2^ = 8.86, *p* = 0.595), and the Cox model had higher predictive power (Harrell’s C-index = 0.702, 95% CI 0.678–0.725, *p* < 0.001).

Statistical analyses were performed using Stata/MP 11.2 (Stata Corp., College Station, TX, USA) and IBM SPSS 25 (IBM SPSS Inc., Chicago, IL, USA). The threshold for statistical significance was set at *p* < 0.05.

## 3. Results

Of the 15,043 participants, 495 (3.3%) developed dementia during the follow-up period, and the mean follow-up period was 51.7 months (SD: 5.15). The mean age of the participants was 73.85 years (SD: 6.27). The non-dementia group’s mean age (73.70, SD: 6.19) was lower than the dementia group’s mean age (78.12, SD: 7.25).

In the univariate analysis, age, education level, health insurance type, BMI, and smoking status were significantly associated with incident dementia (Table 1). As for BMI, the dementia incidence rate was 4.8% in the underweight group, 4.1% in the normal weight group, 2.9% in the overweight group, and 2.5% in the obese group. Thus, as BMI increased, the dementia incidence became significantly lower statistically. In terms of the differences in cardiovascular risk factors between the groups, stroke was more likely to be comorbid in the dementia group than in the comparison group (*p* < 0.001). Additionally, the presence of current smoking was higher in the dementia group (*p* = 0.002), and the number of cardiovascular risk factors was significantly higher in the dementia group than in the comparison group (*p* = 0.003) (Table 2).

The analysis of risk factors for dementia was performed using Cox proportional hazards regression model adjusted for demographic factors (i.e., age, number of years of education), BMI, and smoking status as a lifestyle factor, which were found to be statistically significant in the univariate analysis. Results indicated that the HR for dementia in Medical Aid beneficiaries was 1.77 (95% CI 1.421–2.215), compared with that in health insurance beneficiaries, revealing significantly higher dementia risk in Medical Aid beneficiaries, given that we consider health insurance type as an indicator of economic status. Moreover, the HR for dementia increased when the number of cardiovascular risk factors increased. Compared with participants having no risk factors, dementia risk increased from 1.56 for the presence of three cardiovascular risk factors (HR 1.56, 95% CI 1.071–2.278) to 2.55 for the presence of four cardiovascular risk factors (HR 2.55, 95% CI 1.174–5.546) (Table 3).

## 4. Discussion

In this study, we found that dementia incidence was statistically significantly higher in Medical Aid beneficiaries with lower economic levels and increased as the number of cardiovascular risk factors increased. The present study showed that the risk was higher in Medical Aid beneficiaries than in health insurance subscribers (HR 1.77, 95% CI 1.421–2.215). These results were similar to that of a previous study conducted with 1,161,198 patients and provided elderly patient sample data for the Health Insurance Review and Assessment Service; the study reported that the proportion of Medical Aid beneficiaries among patients with dementia was 15.8%, which was almost twice as high as the proportion among patients without dementia (7.7%) [31].

Among studies on dementia incidence, a three-year longitudinal cohort study of 825 Belgians [32] demonstrated that dementia incidence rate was higher in women with relatively lower socioeconomic status, considered to have low educational levels. According to a systematic review of the literature published from 1985 to 2010 regarding the relationship between education level and dementia [33], 58% of previous studies (51 out of 88 studies) reported that lower education level is a dementia risk factor. In recently published papers, lower economic status and dementia showed a positive relationship in Lebanon (adjusted OR 3.90; 95% CI 1.58–9.60) [34], Japan (OR 1.26; 95% CI 0.80–1.98) [35], and Sweden (HR 1.03; 95% CI 1.01–1.06 in middle-old (65–74 years) population) [36]. Consistent with previous studies, the present study demonstrated that when health insurance type was used as a proxy for lower economic status, Medical Aid beneficiaries had a negligible association with incident dementia upon adjusting for age, education, body mass index, smoking status, and composite cardiovascular disease risk score.

Cardiovascular risk factors have been reported to be important dementia risk factors in previous studies [7,8]. In agreement with this, our results showed that dementia risk increased as the number of cardiovascular risk factors increased. These findings were also consistent with other studies, such as an investigation of elderly people with dementia living in local communities in Korea, which reported that dementia risk increased in people with a history of stroke (7.55 times) or hyperlipidemia (3.66 times) [37], and a survey of people aged 65 years or over in Spain that found that participants with more than one vascular risk factor had a 7.77 times increased risk for vascular dementia compared to those with no vascular risk factors (OR = 7.77; 95% CI 1.55–39.03) [38]. Our findings were also in agreement with a study of dementia risk factors, from 1966 to 2007, which reported that midlife hypertension and hypercholesterolemia, which are risk factors for cardiovascular disease, were associated with a higher risk of developing Alzheimer’s disease later in life [39]. Similarly, a nine-year follow-up study of middle-aged people in the United States found that the dementia risk was 27% higher in participants with one cardiovascular risk factor, 70% higher in those with two cardiovascular risk factors, and twice as high in those with three cardiovascular risk factors, compared with participants with no risk factors [9].

In this study, the number of participants with four cardiovascular risk factors was relatively small (86 of 15,043; 0.5%), while that of three risk factors comprised 5.0% (751 of 15,043) of the study sample. This could be because the participants enrolled and registered for screening and management of dementia in community health centers in Seoul. In other words, because study population was recruited on a community basis, the comorbidity of cardiovascular risk factors was not complex. Therefore, considering the three more prevalent risk factors in the cardiovascular component scale and the economic status, Medical Aid beneficiaries had a 1.77 times higher dementia risk than health insurance subscribers, whereas the presence of three cardiovascular risk factors is associated with a 1.56 times increased dementia risk. This result indicates that economic status is more closely related to dementia risk than cardiovascular risk factors. Therefore, it is necessary to make efforts to reduce health inequality by identifying adults aged 60 years and above with lower economic status as a target population, and providing them with relevant information so that they can receive early treatment through screening and early diagnosis of dementia. In addition, since dementia risk increased significantly with increase in the number of cardiovascular risk factors, there is a need to provide patients with hypertension, diabetes, coronary artery disease, or stroke with adequate treatment and self-care management, as well as education programs, and to monitor them to reduce dementia risk.

This study has several strengths. It used a large community-based prospective cohort and investigated dementia risk in people aged 60 years or over, who were not diagnozed with dementia. Moreover, to the best of our knowledge, this is the first attempt in Korea to investigate dementia risk factors in connection with demographic characteristics, lifestyle factors, health insurance type, and cardiovascular risk factors.

However, this study has some limitations. First, actual income level was not investigated as a marker of economic status; instead, health insurance type was used as a proxy indicator of economic status. Second, dementia incidence was presented as a percentage during the follow-up period because it was not possible to calculate the incidence density rate of dementia per person-year, as the cohort was part of a community-based project and the exact dementia diagnosis date could not be ascertained. Third, dementia types (e.g., Alzheimer’s disease, vascular dementia) were not identified due to the limitations of community-based registry data, because this study was performed using registry data from a public community health center, and socio-demographic variables and cardiovascular risk factors were obtained from self-reported questionnaires rather than from medical records. Finally, although the data analyzed in this study were not relatively recent, it is reasonable to examine the incidence and risk factors of dementia using a community-based screening and management program introduced in Seoul. This initial data on community-based dementia incidence and risk factors could provide information for understanding the temporal trends of dementia incidence and risk factors in South Korea.

## 5. Conclusions

This study showed that social economic status measured by health insurance type and cardiovascular risk factors were associated with dementia incidence in adults aged 60 years or over, residing in local communities in South Korea. Therefore, we propose that public health strategies for dementia prevention and management should be developed and implemented to identify target populations by taking both low social economic status and multiple cardiovascular risk factors into account.

## Figures and Tables

**Table 1:** Sociodemographic characteristics of the participants according to dementia incidence (*n* = 15,043).

Variables	Categories	Total Sample *n* = 15,043		Non-Dementia Group *n* = 14,548 (96.7%)		Incident Dementia Group *n* = 495 (3.3%)		Group Comparison	
								*t* or χ2	*p*
		*n* (%) or *M* (SD)		*n* (%) or *M* (SD)		*n* (%) or *M* (SD)			
Age (years)		73.85	(6.27)	73.70	(6.19)	78.12	(7.25)	−13.38	<0.001
Gender	Female	10,378	(69.0)	10,049	(96.8)	329	(3.2)	1.52	0.217
	Male	4665	(31.0)	4499	(96.4)	166	(3.6)		
Education (years)	0–6	9193	(61.1)	8837	(96.1)	356	(3.9)	25.28	<0.001
	7–12	4418	(29.4)	4311	(97.6)	107	(2.4)		
	≥13	1432	(9.5)	1400	(97.8)	32	(2.2)		
Cohabitation	Living alone	3632	(24.1)	3502	(96.4)	130	(3.6)	1.25	0.263
	Living with someone	11,411	(75.9)	11,046	(96.8)	365	(3.2)		
Type of health insurance	Health insurance beneficiaries	13,488	(89.7)	13,096	(97.1)	392	(2.9)	60.55	<0.001
	Medical Aid beneficiaries	1555	(10.3)	1452	(93.4)	103	(6.6)		
Body mass index (kg/m^2^)	<18.5	544	(3.6)	518	(95.2)	26	(4.8)	27.78	<0.001
	18.5–23	5479	(36.4)	5252	(95.9)	227	(4.1)		
	23–25	4045	(26.9)	3928	(97.1)	117	(2.9)		
	≥25	4975	(33.1)	4850	(97.5)	125	(2.5)		
Drinking status	Non-drinker	12,179	(81.0)	11,765	(96.6)	414	(3.4)	2.38	0.123
	Current drinker	2864	(19.0)	2783	(97.2)	81	(2.8)		
Smoking status	Non-smoker	11,577	(76.9)	11,225	(97.0)	352	(3.0)	10.63	0.005
	Ex-smoker	2310	(15.4)	2219	(96.1)	91	(3.9)		
	Current smoker	1156	(7.7)	1104	(95.5)	52	(4.5)		

**Table 2:** Association between cardiovascular risk factors and dementia incidence (*n* = 15,043).

Cardiovascular Risk Factors	Non-Dementia Group (*n* = 14,548)	Incident Dementia Group (*n* = 495)	Age-Adjusted *p*-Value ^†^
	*n* (%)	*n* (%)	
Cardiovascular risk factors			
Comorbid hypertension	8138 (55.9)	281 (56.8)	0.521
Comorbid diabetes	3011 (20.7)	111 (22.4)	0.210
Comorbid coronary heart diseases	1439 (9.9)	51 (10.3)	0.974
Comorbid stroke	832 (5.7)	64 (12.9)	<0.001
Current smoking	1104 (7.6)	52 (10.5)	0.002
Composite cardiovascular disease risk score			
0	4605 (31.7)	139 (28.1)	Referent
1	6234 (42.9)	204 (41.2)	0.986
2	2916 (20.0)	108 (21.8)	0.284
3	714 (4.9)	37 (7.5)	0.005
4	79 (0.5)	7 (1.4)	0.005
5	0(0)	0(0)	NA

† *p*-values were calculated using a logistic regression model, adjusted for age at the baseline.

**Table 3:** Cox proportional hazards model for the association between health insurance, cardiovascular risk factors, and dementia incidence (*N* = 15,043).

Variables		HR	SE	*p*	95% CI	
Type of health insurance	Health insurance beneficiaries	Referent				
	Medical Aid beneficiaries	1.77 *	0.201	<0.001	1.421	2.215
Composite cardiovascular disease risk score	0	Referent				
	1	0.98	0.109	0.834	0.785	1.216
	2	1.07	0.143	0.588	0.828	1.395
	3	1.56 *	0.301	0.021	1.071	2.278
	4	2.55 *	1.011	0.018	1.174	5.546
C statistic = 0.702, 95% CI = 0.678 to 0.725, *p*-value < 0.001 The Harrell C statistics were obtained by using STATA stcox post-estimation command “estat concordance” and 95% CIs were obtained by using “somersd” package in STATA 11.2						

*Adjusted for age, education, body mass index, and smoking status at the baseline. HR = hazard ratio; SE = standard error; CI = confidence interval.

## References

[1] Ministry of Health and Welfare. https://www.nid.or.kr/info/dataroom_view.aspx?bid=194.

[2] Choi H., Kim S.H., Lee J.-H., Lee A.Y., Park K.W., Lee E.A., Choi S., Na D.L., Jeong J.H. (2018). National responsibility policy for dementia care: Current and future. J. Korean Neurol. Assoc..

[3] Seoul Economy. http://www.sedaily.com/NewsView/1S0XM8HBCZ.

[4] Ministry of Health and Welfare. http://www.mohw.go.kr/react/jb/sjb030301vw.jsp?PAR_MENU_ID=03&MENU_ID=032902&CONT_SEQ=336739&page=1.

[5] Cho M.J. (2009). The prevalence and risk factors of dementia in the Korea elderly. Health Welf. Policy Forum.

[6] Fratiglioni L., Paillard-Borg S., Winblad B. (2004). An active and socially integrated lifestyle in late life might protect against dementia. Lancet Neurol..

[7] Breteler M.M. (2000). Vascular risk factors for Alzheimer’s disease: An epidemiologic perspective. Neurobiol. Aging.

[8] Launer L.J. (2002). Demonstrating the case that AD is a vascular disease: Epidemiologic evidence. Ageing Res. Rev..

[9] Whitmer R.A., Sidney S., Selby J., Johnston S.C., Yaffe K. (2005). Midlife cardiovascular risk factors and risk of dementia in late life. Neurology.

[10] Luchsinger J.A., Tang M.X., Stern Y., Shea S., Mayeux R. (2001). Diabetes mellitus and risk of Alzheimer’s disease and dementia with stroke in a multiethnic cohort. Am. J. Epidemiol..

[11] Tzourio C., Anderson C., Chapman N., Woodward M., Neal B., MacMahon S., Chalmers J., PROGRESS Collaborative Group (2003). Effects of blood pressure lowering with perindopril and indapamide therapy on dementia and cognitive decline in patients with cerebrovascular disease. Arch. Intern. Med..

[12] Stringhini S., Sabia S., Shipley M., Brunner E., Nabi H., Kivimaki M., Singh-Manoux A. (2010). Association of socioeconomic position with health behaviors and mortality. JAMA.

[13] Stern Y. (2002). What is cognitive reserve? Theory and research application of the reserve concept. J. Int. Neuropsychol. Soc..

[14] Okamoto S. (2018). Socioeconomic factors and the risk of cognitive decline among the elderly population in Japan. Int. J. Geriatr. Psychiatry.

[15] Rusmaully J., Dugravot A., Moatti J.P., Marmot M.G., Elbaz A., Kivimaki M., Sabia S., Singh-Manoux A. (2017). Contribution of cognitive performance and cognitive decline to associations between socioeconomic factors and dementia: A cohort study. PLoS Med..

[16] Satizabal C.L., Beiser A.S., Chouraki V., Chene G., Dufouil C., Seshadri S. (2016). Incidence of dementia over three decades in the framingham heart study. N. Engl. J. Med..

[17] Beydoun M.A., Beydoun H.A., Gamaldo A.A., Teel A., Zonderman A.B., Wang Y. (2014). Epidemiologic studies of modifiable factors associated with cognition and dementia: Systematic review and meta-analysis. BMC Public Health.

[18] Lee Y.K., Sung M.R., Lee D.Y. (2011). Comorbidity and health habits of Seoul city elders with dementia. J. Korean Acad. Nurs..

[19] Kim S.M., Seo H.J., Sung M.R. (2014). Factors affecting dementia prevalence in people aged 60 or over: A community based cross-sectional study. J. Korean Acad. Nurs..

[20] Greenland P., Alpert J.S., Beller G.A., Benjamin E.J., Budoff M.J., Fayad Z.A., Foster E., Hlatky M.A., Hodgson J.M., Kushner F.G. (2010). ACCF/AHA guideline for assessment of cardiovascular risk in asymptomatic adults: A report of the American College of Cardiology Foundation/American Heart Association Task Force on Practice Guidelines. Circulation.

[21] Perk J., De Backer G., Gohlke H., Graham I., Reiner Z., Verschuren M., Albus C., Benlian P., Boysen G., Cifkova R. (2012). European guidelines on cardiovascular disease prevention in clinical practice (version 2012). The fifth joint task force of the European society of cardiology and other societies on cardiovascular disease prevention in clinical practice (constituted by representatives of nine societies and by invited experts). Eur. Heart J..

[22] Barzi F., Patel A., Gu D., Sritara P., Lam T.H., Rodgers A., Woodward M., Asia Pacific Cohort Studies Collaboration (2007). Cardiovascular risk prediction tools for populations in Asia. J. Epidemiol. Community Health.

[23] Ueshima H., Sekikawa A., Miura K., Turin T.C., Takashima N., Kita Y., Watanabe M., Kadota A., Okuda N., Kadowaki T. (2008). Cardiovascular disease and risk factors in Asia: A selected review. Circulation.

[24] Lee J.H., Lee K.U., Lee D.Y., Kim K.W., Jhoo J.H., Kim J.H., Lee K.H., Kim S.Y., Han S.H., Woo J.I. (2002). Development of the Korean version of the Consortium to Establish a Registry for Alzheimer’s Disease Assessment Packet (CERAD-K): Clinical and neuropsychological assessment batteries. J. Gerontol. B Psychol. Sci. Soc. Sci..

[25] Kang Y., Na D.L. (2003). Seoul Neuropsychological Screening Battery (SNSB) Manual.

[26] American Psychiatric Association (2013). Diagnostic and Statistical Manual of Mental Disorders (DSM-5®).

[27] Bahk J., Kim Y.Y., Kang H.Y., Lee J., Kim I., Lee J., Yun S.C., Park J.H., Shin S.A., Khang Y.H. (2017). Using the national health information database of the national health insurance service in Korea for monitoring mortality and life expectancy at national and local levels. J. Korean Med. Sci..

[28] Jeon B., Kwon S. (2013). Effect of private health insurance on health care utilization in a universal public insurance system: A case of South Korea. Health Policy.

[29] Kim J.H., Lee K.S., Yoo K.B., Park E.C. (2015). The differences in health care utilization between medical aid and health insurance: A longitudinal study using propensity score matching. PLoS ONE.

[30] Chang W.S., Won K.H., Lee J.Y., Kim E.T., Kweon H.J. (2012). The relationship between obesity and the high probability of dementia based on the body mass index and waist circumference. Korean J. Fam. Med..

[31] Oh J.E. (2015). Direct Medical Costs for Patients with Dementia: Based on Aged Patients Sample for the National Health Insurance Claims Data. Master’s Thesis.

[32] De Deynm P.P., Goeman J., Vervaet A., Dourcy-Belle-Rose B., Van Dam D., Geerts E. (2011). Prevalence and incidence of dementia among 75–80-year-old community-dwelling elderly in different districts of Antwerp, Belgium: The Antwerp Cognition (ANCOG) Study. Clin. Neurol. Neurosurg..

[33] Sharp E.S., Gatz M. (2011). Relationship between education and dementia: An updated systematic review. Alzheimer Dis. Assoc. Disord..

[34] Chaaya M., Phung K., Atweh S., El Asmar K., Karam G., Khoury R.M., Ghandour L., Ghusn H., Assaad S., Prince M. (2017). Socio-demographic and cardiovascular disease risk factors associated with dementia: Results of a cross-sectional study from Lebanon. Prev. Med. Rep..

[35] Nakahori N., Sekine M., Yamada M., Tatsuse T., Kido H., Suzuki M. (2018). A pathway from low socioeconomic status to dementia in Japan: Results from the Toyama dementia survey. BMC Geriatr..

[36] Sundström A., Westerlund O., Kotyrlo E. (2016). Marital status and risk of dementia: A nationwide population-based prospective study from Sweden. BMJ Open.

[37] Kim J.S., Jeong I.S., Kim Y.J., Hwang S.K., Choi B.C. (2003). Screening for high risk population of dementia and development of the preventive program using web. J. Korean Acad. Nurs..

[38] Martinez M.F., Flores J.C., de Las Heras S.P., Lekumberri A.M., Menocal M.G., Imirizaldu J.J.Z. (2008). Risk factors for dementia in the epidemiological study of Munguialde County (Basque Country-Spain). BMC Neurol..

[39] Azad N.A., Al Bugami M., Loy-English I. (2007). Gender differences in dementia risk factors. Gend. Med..

